# Multidimensional EEG mechanisms and explainable prediction of responsiveness to posterior parietal HD-tDCS in chronic disorders of consciousness

**DOI:** 10.3389/fnins.2026.1925503

**Published:** 2026-09-14

**Authors:** Xiangyun Zou, Bin Jiang, Chunyun Zhang

**Affiliations:** 1 Department of Pediatrics, Qilu Hospital of Shandong University (Qingdao), Qingdao, China; 2 Department of Neurosurgery, Qilu Hospital of Shandong University (Qingdao), Qingdao, China

**Keywords:** disorders of consciousness, FOOOF, HD-tDCS, nonlinear EEG, spectral parameterization

## Abstract

Transcranial direct current stimulation (tDCS) is a promising intervention for chronic disorders of consciousness (DoC), yet therapeutic responsiveness varies substantially across patients and does not conform to diagnostic boundaries. Contemporary theories of consciousness implicate the posterior parietal cortex as a critical node for conscious processing, but the electrophysiological mechanisms engaged by posterior parietal stimulation remain poorly characterized. High-definition tDCS (HD-tDCS) was applied over Pz in 42 patients with chronic DoC, who were stratified as responders (R+, *n* = 32) or non-responders (R−, *n* = 10) according to the emergence of new behavioral signs on the Coma Recovery Scale–Revised. Resting-state electroencephalography (EEG) was analyzed using spectral parameterization (FOOOF), band-limited estimates within two frequency windows informed by the ABCD framework (5–9 Hz and 20–35 Hz), recurrence quantification analysis (RQA), Lempel–Ziv complexity (LZc), and normalized spatial complexity (NSC). In R+ only, the aperiodic exponent decreased at four centro-parietal electrodes, power in the 20–35 Hz window increased at nine electrodes, RQA metrics decreased across 13–18 electrodes, LZc increased at 11 electrodes, and NSC in the 20–35 Hz window increased significantly (*p* = 0.0222). No corresponding changes were detected in R−. Relief-based feature selection identified the aperiodic exponent and the 20–35 Hz NSC feature from eight candidate biomarkers for training a Bayesian-optimized support vector machine. SHapley Additive exPlanations indicated comparable contributions from both features. These findings identify responder-specific changes in periodic and aperiodic spectral parameters and in nonlinear EEG descriptors following Pz-targeted HD-tDCS. They are consistent with modulation of cortical state and large-scale dynamics, but do not by themselves demonstrate excitation–inhibition rebalancing or thalamocortical loop reinforcement, and the single-arm design without sham stimulation precludes attribution of the observed changes specifically to HD-tDCS.

## 1 Introduction

Chronic disorders of consciousness (DoC) are prolonged states of impaired awareness lasting more than 4 weeks after severe brain injury, most commonly classified as vegetative state/unresponsive wakefulness syndrome (VS/UWS), minimally conscious state (MCS), or emergence from MCS (EMCS) (Aubinet et al., 2025; Giacino et al., 2014). The Coma Recovery Scale–Revised (CRS-R) remains the most widely used standardized behavioral instrument for differentiating these states, but behavioral assessment is constrained by fluctuating arousal, impaired motor output, and aphasia, contributing to reported misdiagnosis rates of up to 40% (Annen et al., 2019). In this context, transcranial direct current stimulation (tDCS) has emerged as a promising non-invasive neuromodulatory intervention (Wang et al., 2020; Kuo et al., 2013). However, treatment efficacy varies substantially across individuals and does not respect diagnostic boundaries: some patients diagnosed with VS/UWS show measurable behavioral improvement after stimulation, whereas a substantial proportion of patients with MCS show no detectable response (Martens et al., 2020). This discordance indicates that diagnosis-based stratification alone is insufficient for individualized treatment decisions. Objective electrophysiological biomarkers capable of predicting stimulation responsiveness at the individual level are therefore needed, motivating a shift from diagnostic labels toward a responder (R+) versus non-responder (R−) stratification framework. The present study aims to identify electrophysiological biomarkers of HD-tDCS responsiveness at Pz by combining spectral parameterization, nonlinear dynamical analysis, and explainable machine learning.

Principled selection of a neuromodulatory target requires engagement with contemporary theories of consciousness. Despite ongoing debate regarding the role of the prefrontal cortex, three influential frameworks—Integrated Information Theory (IIT), Global Neuronal Workspace Theory (GNW), and Recurrent Processing Theory (RPT)—converge on the importance of posterior cortical regions for conscious processing (Xu et al., 2024; Guo et al., 2025; Marshall et al., 2016; Mashour et al., 2020; Schiff, 2023). IIT assigns high integrated causal structures to posterior cortical areas; RPT emphasizes recurrent feedback within sensory and posterior association cortices; and GNW, while traditionally emphasizing fronto-parietal broadcasting, increasingly recognizes the indispensable contribution of posterior nodes. This convergence underpins the “posterior hot zone” hypothesis, which identifies the parieto-occipito-temporal junction, including the precuneus and posterior cingulate cortex, as a candidate network for the generation and maintenance of conscious content (Tononi et al., 2016). This theoretical consensus provides a network-level rationale for targeting Pz, a scalp site that most closely approximates the posterior parietal and precuneal midline network.

Despite strong theoretical support for posterior cortical regions, existing tDCS and high-definition tDCS (HD-tDCS) studies in DoC have predominantly targeted the left dorsolateral prefrontal cortex (DLPFC; F3) (Mensen et al., 2020; Hermann et al., 2020). Although several trials have reported CRS-R improvements following DLPFC stimulation, the posterior parietal target remains comparatively underexplored: only isolated pilot studies have investigated precuneus-directed HD-tDCS, and the first prospective comparison of F3, Pz, and sham stimulation appeared recently (Guo et al., 2025). This mismatch between theoretical relevance and limited empirical evidence defines an important research gap. HD-tDCS, with its 4 × 1 ring-electrode configuration, offers greater spatial focality than conventional large-pad tDCS, reducing diffuse current spread and strengthening target-effect attribution (Han et al., 2022). This technical advantage makes HD-tDCS particularly suitable for probing the specific contribution of Pz to consciousness modulation.

Characterizing the electrophysiological mechanisms of posterior parietal stimulation requires analytical tools that go beyond conventional frequency-band power analysis. Traditional approaches conflate periodic oscillatory activity with the aperiodic 1/f background, potentially misattributing changes in spectral slope to oscillatory power shifts (McKeown et al., 2024). The FOOOF (Fitting Oscillations and One-Over-F) spectral parameterization model addresses this confound by decomposing the power spectrum into independent periodic peaks and an aperiodic component (Gerster et al., 2022; Drążyk et al., 2024; Donoghue et al., 2020). The aperiodic exponent has been proposed as a mesoscale proxy for cortical excitation–inhibition (E/I) balance, although it remains an indirect electrophysiological correlate rather than a direct physiological measurement (Gao et al., 2017). Conventional frequency bands are defined empirically and do not map directly onto specific neural circuits. Disruption of corticothalamic circuits is widely regarded as a central pathophysiological mechanism of impaired consciousness after severe brain injury (Edlow et al., 2021). The ABCD framework is a categorical model of the complete resting EEG power spectrum rather than a naming system for individual frequency bands (Schiff, 2016; Comanducci et al., 2020; Forgacs et al., 2017). A-type spectra are dominated by very slow activity near 1 Hz, B-type spectra show a dominant theta-range peak, C-type spectra show coexisting theta- and beta-range components, and D-type spectra show alpha- and beta-range components. These profiles have been interpreted as reflecting progressively greater corticothalamic functional integrity. Comanducci et al., (2020) annotate the frequency range associated with each profile, based on Schiff, (2016): A, below 1 Hz; B, approximately 5–9 Hz; C, the same low-frequency component together with approximately 20–35 Hz; and D, approximately 9–12 Hz together with approximately 20–35 Hz. The present study uses these two annotated ranges as hypothesis-driven operational windows. They are not themselves ABCD categories, and neither window can independently establish a transition between ABCD states, which is defined by the joint configuration of the whole spectrum.

Even after spectral parameterization, frequency-domain analyses cannot fully capture the nonlinear dynamical properties of neural activity. Higher awareness states are associated with richer and less predictable activity, whereas low-consciousness states such as VS/UWS are characterized by more rigid and repetitive patterns (Liu et al., 2023; Wang et al., 2022). Recurrence quantification analysis (RQA) characterizes the recurrence and determinism of neural trajectories through phase-space reconstruction (Marwan et al., 2007). Lempel–Ziv complexity (LZc) quantifies the diversity of symbolic subsequences in a discretized signal and has been used to characterize changes in EEG complexity across altered states and levels of consciousness, although estimates depend on preprocessing, binarization and normalization choices (Liu et al., 2023; Schartner et al., 2015). Normalized spatial complexity (NSC) complements these temporal metrics by estimating large-scale spatial differentiation across electrode signals using principal-component eigenvalue entropy (Jia et al., 2018).

The clinical value of multidimensional EEG features ultimately depends on whether they can be integrated into an actionable prediction tool. Machine learning has increasingly been applied to EEG-based classification, but existing models in DoC have focused primarily on diagnostic categorization rather than prediction of neuromodulatory responsiveness (Wang et al., 2025). In high-risk, small-sample clinical settings such as DoC, a model that cannot explain the basis of its decisions is unlikely to gain clinical acceptance. The SHAP (SHapley Additive exPlanations) framework, grounded in cooperative game theory, quantifies the contribution of each feature to individual predictions, providing both global feature-importance rankings and patient-level attribution (Lundberg and Lee, 2017). This transparency helps bridge data-driven prediction and clinical trustworthiness by generating outputs that are not only accurate but also interpretable and verifiable by clinicians (Wang et al., 2025).

The present study revisits this cohort because the original report (Zhang et al., 2022) examined conventional frequency-band power, functional connectivity, and normalized spatial complexity, but did not separate periodic from aperiodic spectral components, did not apply nonlinear dynamical measures such as RQA or LZc, and did not attempt prediction of treatment responsiveness. These complementary analyses can be performed on the same resting-state EEG recordings without additional data collection, and retaining the identical cohort avoids introducing confounds from differences in clinical protocol or acquisition setup.

In summary, three interconnected research gaps remain. First, although contemporary theories of consciousness identify the posterior parietal cortex as a critical node, systematic electrophysiological evidence for the mechanisms of Pz-targeted HD-tDCS is limited. Second, EEG studies of neuromodulation in DoC have largely examined single analytical dimensions. Third, no established computational framework translates such multidimensional features into an explainable model for predicting responsiveness. Building on our previously published analysis of this cohort (Zhang et al., 2022), the present secondary analysis asks whether separating periodic activity from the aperiodic background, and examining hypothesis-driven spectral and nonlinear descriptors, provides information complementary to the previously reported conventional spectral, network and NSC findings. The contributions claimed here are the FOOOF-derived periodic–aperiodic analysis, the evaluation of the two ABCD-informed frequency windows, the nonlinear dynamical measures (RQA and LZc), and their integration within an explainable prediction framework. The cohort, the behavioral outcomes, the conventional-band analyses and the NSC method itself were reported previously and are not presented as novel.

### 1.1 Study provenance and relationship to prior work

This study is a secondary analysis of the clinical and EEG dataset reported by Zhang et al. (2022). In that study, 45 patients with prolonged disorders of consciousness were recruited at the Fifth Affiliated Hospital of Zhengzhou University and Zhengzhou Central Hospital, Zhengzhou University, between October 2016 and October 2020, and all received the 14-day Pz-targeted HD-tDCS protocol; three were excluded from the electrophysiological analysis because of poor EEG signal quality, leaving 42 participants. The original study was approved by the Ethics Committees of the Fifth Affiliated Hospital of Zhengzhou University (KY2020024) and Zhengzhou Central Hospital, Zhengzhou University (201614), and written informed consent was obtained from the legal guardians of all participants. The present secondary analysis was approved by the Ethics Committee of Qilu Hospital of Shandong University (Qingdao) (approval no. KYLL-KS-2025153). The study design and analysis workflow are summarized in Figure 1.

**Figure 1:**
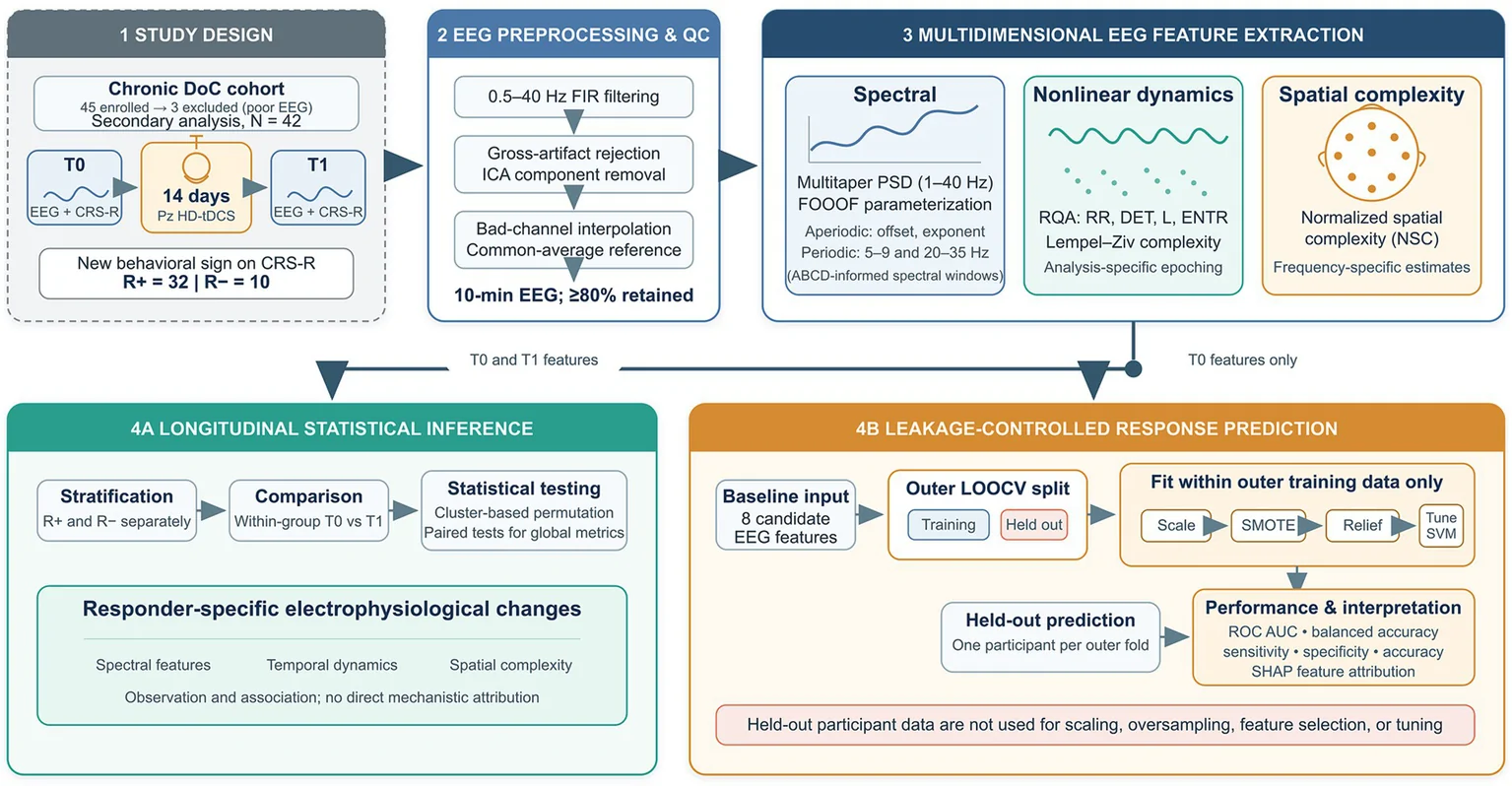
Study design and analysis workflow. Of 45 patients enrolled in the previously reported cohort (Zhang et al., 2022), three were excluded from the EEG analysis because of poor signal quality, leaving 42. Resting-state EEG and CRS-R assessment were obtained before (T0) and after (T1) 14 days of Pz-targeted HD-tDCS, and participants were classified as responders (R+) or non-responders (R−). The predictive branch used pre-stimulation features only. Dashed borders, components reported previously; solid borders, analyses conducted here. Figure structure follows Varun et al. (Varun et al., 2026). HD-tDCS, high-definition transcranial direct current stimulation; CRS-R, Coma Recovery Scale–Revised; EEG, electroencephalography; R+, responders; R−, non-responders; T0, pre-stimulation; T1, post-stimulation; FOOOF, Fitting Oscillations and One-Over-F; RQA, recurrence quantification analysis; LZc, Lempel–Ziv complexity; NSC, normalized spatial complexity; SVM, support vector machine; SHAP, SHapley Additive exPlanations.

### 1.2 Participants and study design

Inclusion criteria were: (1) a clinical diagnosis of vegetative state/unresponsive wakefulness syndrome (VS/UWS) or minimally conscious state (MCS) persisting for at least 3 months after brain injury; (2) medical stability without acute complications; and (3) a stable level of consciousness during the month before enrollment. Exclusion criteria were: (1) metallic implants or electronic devices within the cranium; (2) a history of epilepsy or seizure activity within the preceding month; (3) extensive cranial defects or open skull fractures; (4) use of medications known to substantially alter cortical excitability; and (5) unstable vital signs.

Of the 42 participants included in the present analysis, 28 were male and 14 female, with a mean age of 51.55 ± 15.3 years. Participant-level demographic and clinical characteristics, including diagnosis and all six CRS-R subscale scores at both time points, are provided in Supplementary Table S2.

### 1.3 HD-tDCS protocol

Stimulation was performed in the Pz region using a transcranial electrical stimulator (Soterix Medical Inc., New York, NY, United States) with a 4 × 1 ring-electrode configuration. The anodal electrode was placed at Pz, corresponding to the posterior parietal cortex in the International 10–20 system. Four cathodal return electrodes were arranged symmetrically around the target site at an approximate distance of 3.5 cm from the center.

Stimulation was delivered at a constant current intensity of 2 mA for 20 min per session, with 30-s linear ramp-up and ramp-down periods. Each participant received two sessions per day, separated by at least 4 h, for 14 consecutive days, yielding 28 stimulation sessions in total. Conductive electrode gel was applied to ensure adequate scalp–electrode contact, and impedance was maintained below 5 kΩ throughout each session.

### 1.4 Clinical assessment

Consciousness level was assessed using the CRS-R, a standardized clinical instrument comprising six subscales: auditory, visual, motor, oromotor/verbal, communication, and arousal functions. The CRS-R was administered by trained clinicians at two time points: within 2 days before the first stimulation session (pre-stimulation, T0) and within 2 days after the final stimulation session (post-stimulation, T1). Responder status was defined *a priori* as the emergence at T1 of at least one behavioral sign of consciousness on the CRS-R that was absent at T0, assessed against the standardized CRS-R diagnostic criteria. A numerical increase in total CRS-R score that did not meet this item-level criterion was not sufficient for classification as a responder. The same criterion was applied in the previous report on this cohort (Zhang et al., 2022). Participant-level subscale scores at both time points are provided in Supplementary Table S2 so that every classification can be verified.

### 1.5 EEG data acquisition

Resting-state electroencephalography (EEG) was recorded for 20 min using a Boruikang 32-channel amplifier with 30 scalp electrodes placed according to the International 10–20 system; the full montage is listed in Supplementary Methods §2. Signals were sampled at 1,000 Hz and electrode impedances were maintained below 5 kΩ. Recordings were obtained during behaviorally monitored wakeful rest. The CRS-R arousal-facilitation protocol was applied immediately before acquisition where necessary, and any recording in which the participant closed the eyes for more than a few seconds, or in which sleep features such as spindles or K-complexes appeared, was discarded and repeated. EEG was acquired at two time points: pre-stimulation (T0) and post-stimulation (T1, after 14 days of HD-tDCS).

### 1.6 EEG preprocessing

All EEG data were preprocessed offline using the EEGLAB toolbox in MATLAB 2024b (MathWorks, Natick, MA, United States) (Delorme and Makeig, 2004). Processing steps were applied in the following order: band-pass FIR filtering (0.5–40 Hz), notch filtering (48–52 Hz), manual rejection of grossly artifactual segments, identification of bad channels by two experienced neuroelectrophysiologists with spherical-spline interpolation, independent component analysis for removal of ocular, cardiac and myogenic components, and re-referencing to the common average. Datasets retaining less than 80% of the recording were treated as invalid. The spectral analyses were performed on the epoched dataset at the native sampling rate; the nonlinear analyses used data resampled to 100 Hz and segmented into 5-s epochs with 2-s overlap. All numerical parameters and software versions are given in Supplementary Methods §2.

### 1.7 Spectral parameterization and aperiodic analysis

Power spectral analysis was performed using FieldTrip (Donders Institute, Nijmegen, The Netherlands) in MATLAB 2024b (Oostenveld et al., 2011). Power spectral density was estimated per electrode using the multitaper method with DPSS (Slepian) tapers (cfg.method = ‘mtmfft’; cfg.foilim = [1, 39] Hz; cfg.tapsmofrq = 4), computed per epoch and averaged within participant and channel, giving a frequency resolution of 0.5 Hz. In FieldTrip this smoothing setting corresponds to a ± 4 Hz kernel, which is suited to broadband characterization but limits the resolution of narrow or closely spaced spectral features; the 5–9 Hz window is 4 Hz wide and therefore falls within a single kernel. Spectral parameterization was then performed using the Fitting Oscillations and One-Over-F (FOOOF) algorithm within a FieldTrip-compatible pipeline, decomposing the power spectral density into an aperiodic (1/f-like) component and a periodic component (Donoghue et al., 2020). Fitting used the default settings of the underlying algorithm, which are listed in full in Supplementary Table S3.

The aperiodic component was characterized by two parameters: offset and exponent (slope). The aperiodic exponent reflects the steepness of spectral decay and has been proposed as a mesoscale proxy for cortical excitation–inhibition (E/I) balance; a reduced slope, or flatter spectrum, is associated with increased cortical excitability (Gao et al., 2017). Pre- to post-stimulation changes in the aperiodic exponent were computed for each electrode.

### 1.8 ABCD-informed frequency windows

Building on the spectral parameterization results, band-limited power was quantified within two frequency windows informed by the ABCD framework: 5–9 Hz and 20–35 Hz. These ranges correspond to the B-type profile (Schiff, 2016; Comanducci et al., 2020) and to the faster component shared by the C- and D-type profiles, respectively. Because A–D denote whole-spectrum profiles rather than individual bands, the two intervals are referred to throughout as the ABCD-informed low-frequency window and the ABCD-informed higher-frequency window rather than by any A–D label.

For each electrode, band-limited values were computed as the mean of the fitted FOOOF model spectrum across the frequency bins of each window and therefore include the aperiodic component. An aperiodic-corrected version of the same analysis, together with equivalent estimates in the canonical delta, theta, alpha, beta and gamma ranges, is reported in Supplementary Table S4. The windows are hypothesis-driven operational features: they do not constitute ABCD categories, and scalp-level power cannot localize a generator or establish thalamocortical connectivity. Pre- and post-stimulation values were compared for each electrode.

### 1.9 Nonlinear EEG analysis

To characterize the effects of Pz-targeted HD-tDCS on neural dynamics beyond the spectral domain, we computed nonlinear EEG features including recurrence quantification analysis (RQA) and Lempel–Ziv complexity (LZc). Preprocessed continuous EEG data were first downsampled to 100 Hz to reduce computational burden while preserving the relevant temporal dynamics. The downsampled signal at each electrode was then segmented into 5-s epochs with a 2-s overlap between adjacent segments (Wang et al., 2022). All nonlinear metrics were computed for each segment and averaged across segments to yield a single value per electrode per participant.

#### 1.9.1 Recurrence quantification analysis (RQA)

RQA was performed to assess the deterministic structure of the EEG time series. Phase-space reconstruction used embedding dimension m = 3, time delay τ = 1 sample, a fixed recurrence threshold ε = 0.5 and the Euclidean norm, adopted from prior applications of RQA to EEG rather than optimized on the present dataset (Supplementary Methods §5). Four RQA metrics were extracted: recurrence rate (RR), determinism (DET), average diagonal line length (L), and Shannon entropy of the diagonal line length distribution (ENTR) (Marwan et al., 2007).

#### 1.9.2 Lempel–Ziv complexity (LZc)

Lempel–Ziv complexity was computed to quantify the temporal richness of EEG signals. Each 5-s epoch was binarized at its own mean amplitude, and complexity was computed on the resulting 500-sample binary sequence (Lempel and Ziv, 1976), so that all estimates derive from sequences of identical length; epoch values were averaged within channel and participant. The implementation and the normalization used are specified in Supplementary Methods §5. Higher LZc values indicate a greater rate of novel pattern generation in the time series.

### 1.10 Normalized spatial complexity (NSC)

Normalized spatial complexity (NSC) was computed to quantify how evenly signal variance is distributed across the scalp. For each band, data were band-pass filtered and principal component analysis was applied to the channel × time matrix across all 30 channels; NSC was taken as the Shannon entropy of the normalized eigenvalue distribution divided by the natural logarithm of the number of retained components, so that values approach 0 when variance is concentrated in one spatial component and 1 when it is evenly distributed (Supplementary Methods §6). Values were computed per epoch and averaged within participant, separately for the two ABCD-informed windows (Jia et al., 2018). The measure was applied to this cohort across the canonical bands in the earlier report on these data (Zhang et al., 2022); the two ABCD-informed windows are analyzed here for the first time.

### 1.11 Machine learning classification

To translate multidimensional EEG features into a clinically applicable predictive framework, we developed a machine learning pipeline to classify patients as responders (R+) or non-responders (R−) using pre-stimulation EEG features. Eight EEG features measured at Pz or at the global level were evaluated: (1) Pz aperiodic exponent; (2) 20–35 Hz band-limited power; (3) determinism (DET); (4) average diagonal line length (L); (5) recurrence rate (RR); (6) Shannon entropy (ENTR); (7) mean Lempel–Ziv complexity (LZc_mean); and (8) 20–35 Hz normalized spatial complexity (NSC). The analysis used a nested resampling design with a leave-one-participant-out outer loop and an inner stratified cross-validation for model selection. Standardization, handling of class imbalance, Relief-based feature ranking, choice of the number of retained features and Bayesian optimization of the support-vector-machine hyperparameters were all performed using only the outer training participants, and the fitted pipeline was applied once to the held-out participant. Performance was computed from the pooled out-of-fold predictions. The Relief-selected feature set and the full eight-feature set were compared under the identical framework. Hyperparameter search spaces and the complete pipeline are given in Supplementary Methods §8. Model interpretability was assessed using the SHapley Additive exPlanations (SHAP) framework (Lundberg and Lee, 2017).

### 1.12 Statistical analysis

All statistical analyses were performed using MATLAB 2024b with the FieldTrip toolbox. Electrode-level metrics were compared between pre- and post-stimulation conditions using cluster-based permutation tests with dependent-samples t-tests at each electrode, spatially contiguous clustering by electrode adjacency, and the maximum sum of t-values within a cluster as the cluster-level statistic. The cluster-forming threshold was α = 0.001, the reference distribution was generated from 5,000 permutations of the condition labels, and tests were two-tailed. Adjacency was derived from the two-dimensional electrode layout using ft_prepare_neighbours, with no minimum number of neighboring channels required. Inference is at the level of the cluster rather than the individual electrode, so electrode identities are reported descriptively to characterize cluster extent, and cluster-level statistics with Monte Carlo *p* values are given.

For the NSC analysis, pre- to post-stimulation differences were assessed using paired t-tests for each of the seven frequency bands. Multiple comparisons were controlled using the Benjamini–Hochberg false discovery rate (FDR) procedure. The significance level was set at α = 0.05 (two-tailed) for all analyses.

## 2 Results

### 2.1 Clinical outcomes and group classification

Participant-level demographic and clinical characteristics are summarized in Supplementary Table S2.

Based on the prespecified CRS-R response criterion described in Section 1.4, patients were classified as responders (R+, *n* = 32) or non-responders (R−, *n* = 10). In the R+ group, mean CRS-R total score increased significantly from 8.26 ± 3.52 at baseline (T0) to 11.07 ± 5.16 at post-stimulation (T1; paired *t*-test, *p* = 0.007). No significant CRS-R improvement was observed in the R− group. All subsequent EEG analyses were conducted separately for R+ and R−.

### 2.2 Aperiodic spectral changes

The aperiodic exponent (1/f slope) was extracted from the FOOOF spectral parameterization model and compared between pre- and post-stimulation conditions using cluster-based permutation paired *t*-tests (Figure 2A).

**Figure 2:**
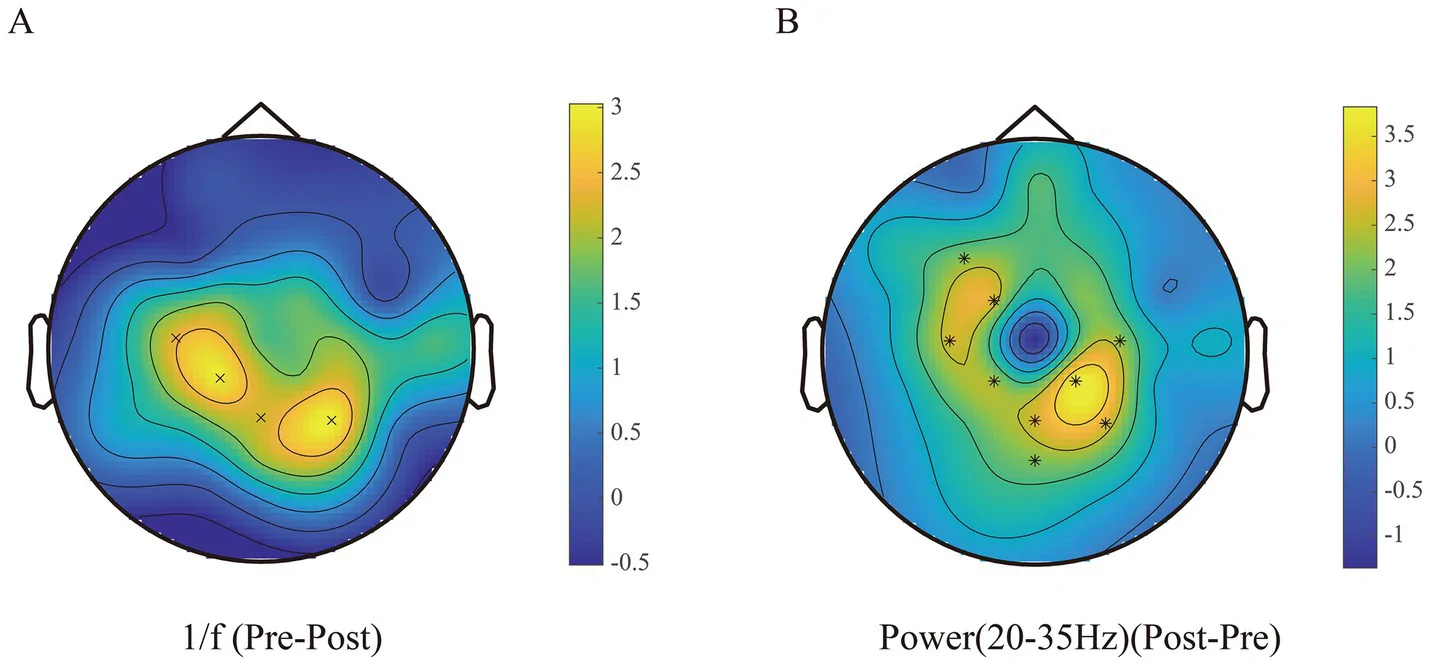
Topographic distribution of spectral changes in the responder group (R+). **(A)** Aperiodic exponent. **(B)** Band-limited power in the 20–35 Hz window. Panel A shows the pre-minus-post contrast, whereas panel B shows the post-minus-pre contrast; warmer colors indicate larger positive values of the respective contrast; markers denote electrodes contributing to the significant cluster. Inference is at the cluster level, and electrode identities describe cluster extent. No cluster was detected in R−. R+, responders; R−, non-responders.

In the R+ group, a significant negative cluster was identified for the aperiodic exponent, comprising four electrodes (C3, P4, Pz, and CP1) over left central and midline parietal regions. Consistent with the inferential scope of cluster-based permutation testing, the electrodes listed describe the spatial extent of the cluster rather than constituting the units of inference.

No significant pre- to post-stimulation changes in the aperiodic exponent were detected at any electrode in the R− group.

### 2.3 Band-limited power changes in the 20–35 Hz window

Band-limited power within the ABCD-informed higher-frequency window (20–35 Hz) was compared between pre- and post-stimulation conditions using cluster-based permutation paired t-tests (Figure 2B).

In the R+ group, a significant positive cluster was identified comprising nine electrodes (F3, C3, C4, P4, Pz, FC1, CP1, CP2, and POz), extending from central to parieto-occipital regions. Corresponding analyses in the 5–9 Hz window and in the canonical frequency bands are reported in Supplementary Table S4.

Four electrodes (C3, P4, Pz, and CP1) contributed to clusters in both the aperiodic exponent and the 20–35 Hz analyses.

No cluster survived in the R− group.

### 2.4 Nonlinear EEG dynamics: recurrence quantification analysis

RQA was performed to assess stimulation-induced changes in the deterministic structure of neural dynamics. Four RQA metrics—recurrence rate (RR), determinism (DET), average diagonal line length (L), and Shannon entropy of the diagonal line length distribution (ENTR)—were compared between pre- and post-stimulation conditions using cluster-based permutation paired t-tests (Figure 3).

**Figure 3:**
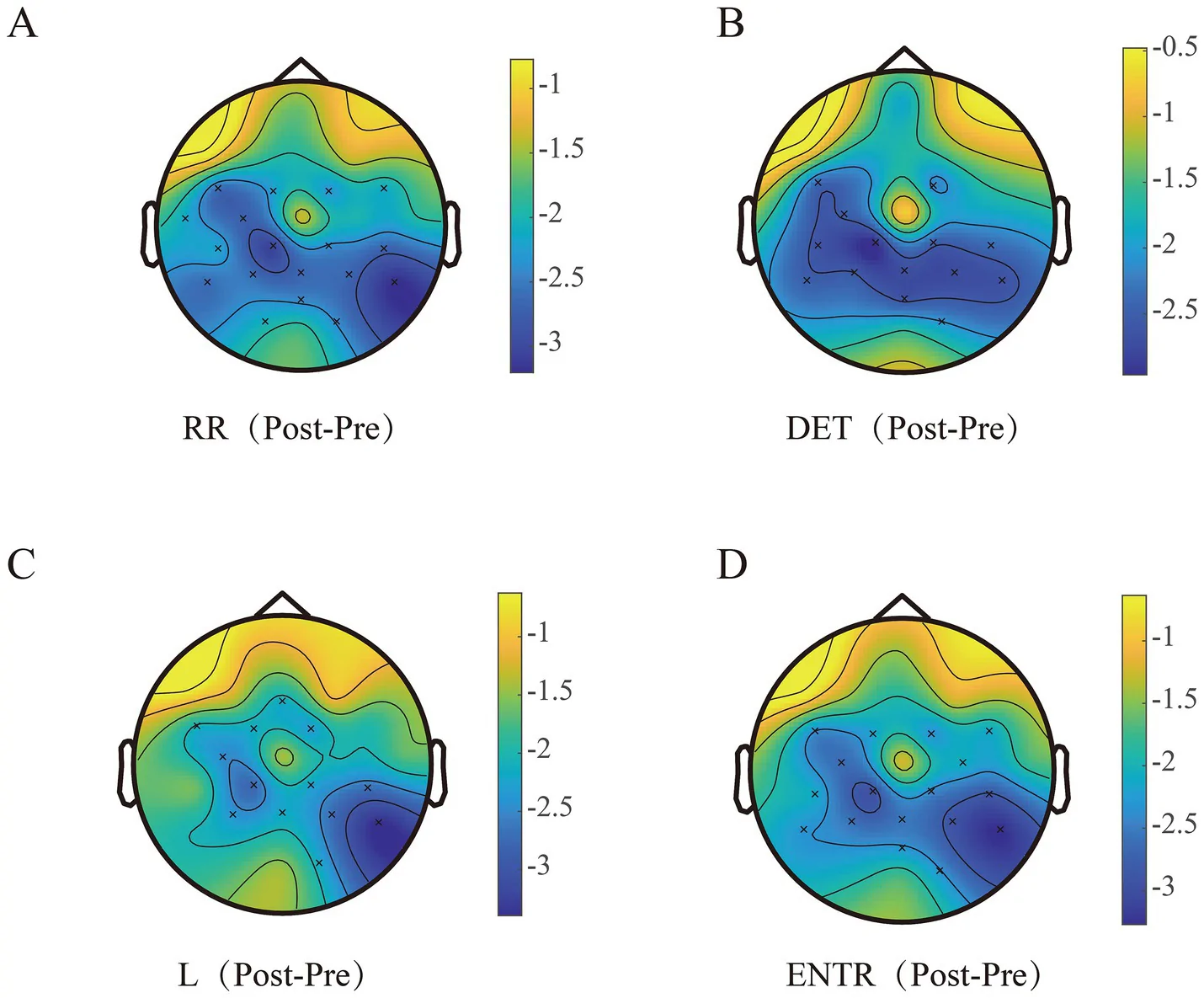
Topographic maps of recurrence quantification analysis (RQA) changes in the responder group (R+). Color maps show *t* values for the post − pre difference; cooler colors indicate decreases. Markers denote electrodes contributing to significant clusters (cluster-based permutation paired *t*-tests, 5,000 permutations, two-tailed). **(A)** Recurrence rate, 18 electrodes. **(B)** Determinism, 14. **(C)** Average diagonal line length, 13. **(D)** Shannon entropy of the diagonal line length distribution, 17. Electrode identities describe cluster extent. No significant change was detected in R−. Abbreviations: RQA, recurrence quantification analysis; R+, responders; R−, non-responders; RR, recurrence rate; DET, determinism; L, average diagonal line length; ENTR, Shannon entropy of diagonal line length distribution.

In the R+ group, all four RQA metrics decreased after stimulation across widespread scalp regions: recurrence rate across 18 electrodes, determinism across 14, average diagonal line length across 13, and diagonal-line entropy across 17. Electrode identities describe the spatial extent of each cluster and are shown in Figure 3.

Across all four RQA metrics, seven electrodes contributed to significant clusters: C3, CP1, CP2, CP6, P4, P8, and Pz. These electrodes lie predominantly over centro-parietal regions surrounding the stimulation site.

No significant pre- to post-stimulation changes in any RQA metric were detected in the R− group.

### 2.5 Temporal complexity: Lempel–Ziv complexity

Mean Lempel–Ziv complexity (LZc_mean) was computed to quantify stimulation-induced changes in the temporal richness of EEG signals. Pre- to post-stimulation comparisons were performed using cluster-based permutation paired *t*-tests (Figure 4A).

**Figure 4:**
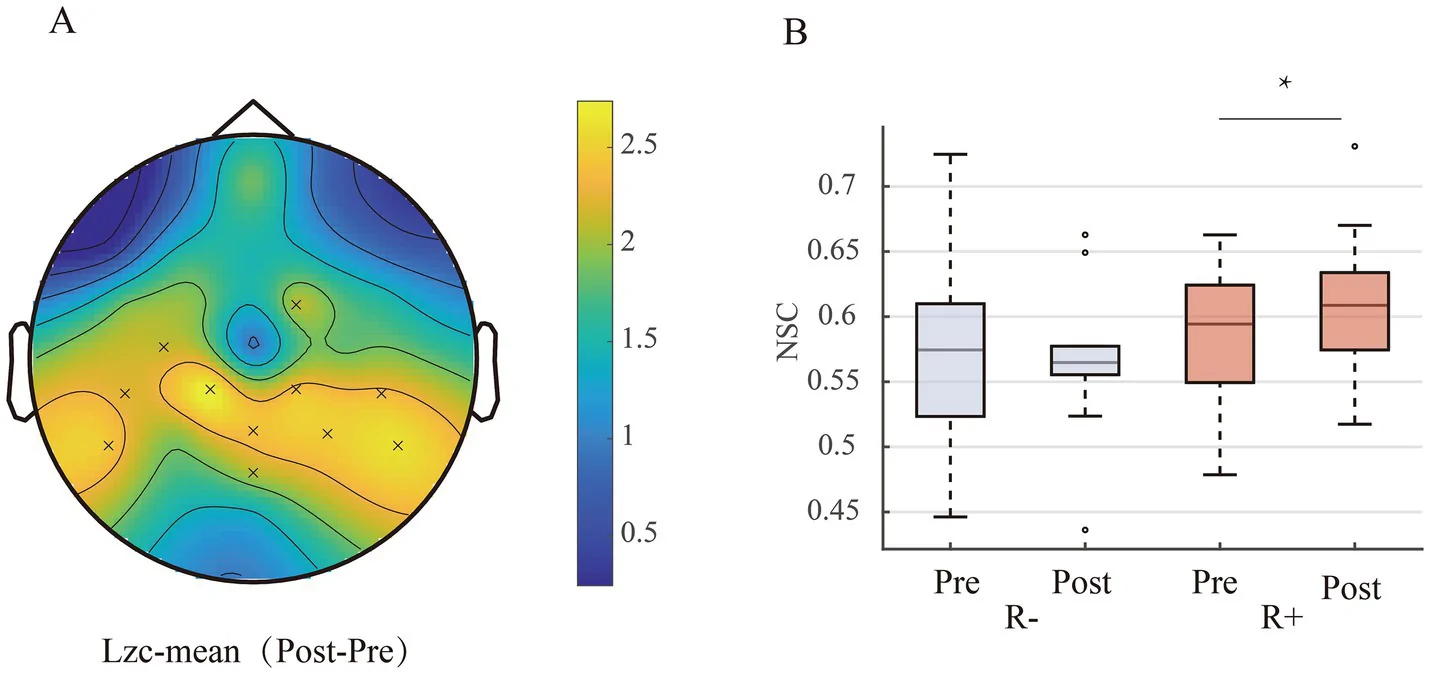
Temporal and spatial complexity in the responder group (R+). **(A)** Topographic map of Lempel–Ziv complexity; markers denote electrodes contributing to the significant cluster. **(B)** Normalized spatial complexity in the 20–35 Hz window before and after stimulation. LZc, Lempel–Ziv complexity; NSC, Normalized spatial complexity; R+, Responders; R−, Non-responders.

In the R+ group, a significant cluster included 11 electrodes showing increased LZc_mean: C3, P4, P7, P8, Pz, FC2, CP5, CP1, CP2, CP6, and POz (cluster-level *p* < 0.05). The electrodes contributing to this cluster lie over centro-parietal and parieto-occipital regions and overlap those identified in the RQA analysis. The direction of change was opposite to that of the RQA metrics, which decreased.

No significant changes in LZc_mean were detected in the R− group.

### 2.6 Spatial complexity: normalized spatial complexity

Normalized spatial complexity (NSC) was computed within the ABCD-informed higher-frequency window (20–35 Hz) and compared between pre- and post-stimulation conditions using paired t-tests with false discovery rate correction across the seven frequency bands (Figure 4B).

In the R+ group, NSC in the 20–35 Hz window increased significantly after stimulation (*p* = 0.0222). This finding overlaps the beta2-band (20–30 Hz) NSC increase previously reported for this cohort by Zhang et al. (2022), and the two results are therefore related rather than independent.

No significant change in NSC was observed at any frequency band in the R− group.

### 2.7 Machine learning classification of stimulation responsiveness

Eight candidate pre-stimulation features were evaluated within the nested validation framework described in Section 1.11 (Figure 5). Relief ranking identified the aperiodic exponent and the 20–35 Hz NSC feature as the two highest-ranked features. Performance metrics for the Relief-selected model and for the full eight-feature model were computed from the pooled out-of-fold predictions. At the selected operating threshold, the Relief-selected model achieved a sensitivity of 85.7% and a specificity of 75.0%, corresponding to a balanced accuracy of 80.4%. The area under the receiver operating characteristic curve was 0.786. Given the small sample size and the absence of external validation, these performance estimates should be regarded as exploratory.

**Figure 5:**
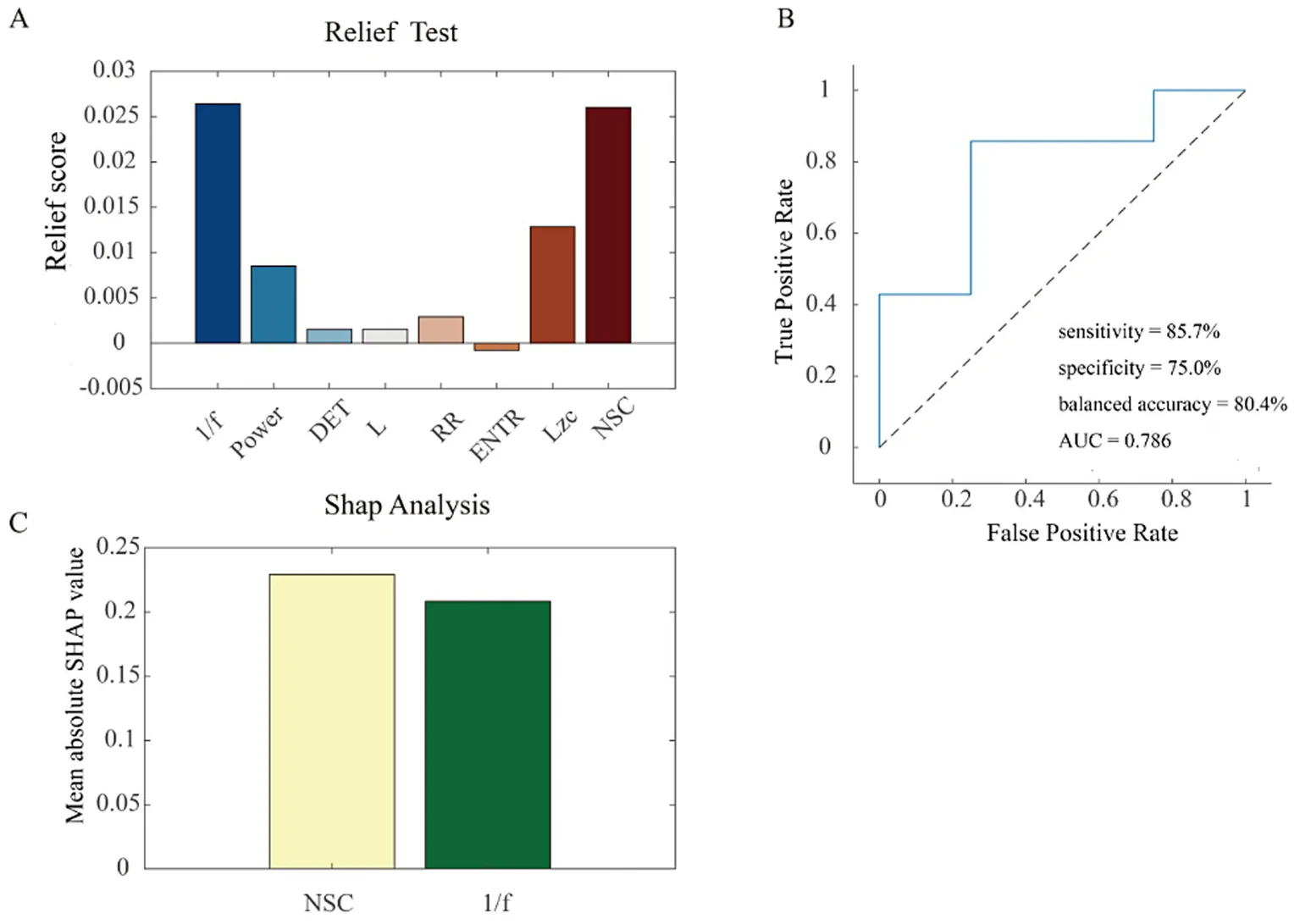
Prediction of HD-tDCS responsiveness. **(A)** Relief weight for each candidate feature. **(B)** Receiver operating characteristic curve; axes are labeled false positive rate (1 − specificity) and true positive rate (sensitivity). **(C)** Mean absolute SHAP value for each retained feature. HD-tDCS, high-definition transcranial direct current stimulation; SHAP, SHapley Additive exPlanations; NSC, normalized spatial complexity; ROC, receiver operating characteristic.

Model interpretability was assessed using SHAP. The mean absolute SHAP values indicated that the 20–35 Hz NSC feature and the aperiodic exponent contributed comparably to the model’s classification decisions (Figure 5C).

## 3 Discussion

This secondary analysis examined the electrophysiological correlates of Pz-targeted HD-tDCS in chronic disorders of consciousness by combining spectral parameterization, two ABCD-informed frequency windows, nonlinear dynamics, spatial complexity and explainable machine learning within a responder-based stratification framework. The principal observations were: (1) the aperiodic exponent decreased and 20–35 Hz band-limited power increased in responders only; (2) RQA metrics decreased and Lempel–Ziv complexity increased in responders only; (3) NSC in the 20–35 Hz window increased in responders only; and (4) two pre-stimulation features supported above-chance discrimination between responders and non-responders. All EEG measures used here are indirect descriptors of scalp-recorded population activity, and we set out their interpretive limits before discussing what they may reflect. A change in the aperiodic exponent may be compatible with altered population-level excitation and inhibition, but it is not a direct or unique measure of synaptic E/I balance. The two frequency windows summarize spectral components that overlap ranges emphasized in the ABCD framework; they do not by themselves classify an ABCD state, and scalp-level power cannot localize a thalamic generator or establish thalamocortical connectivity. RQA, LZc and NSC quantify temporal recurrence, symbolic diversity and the distribution of spatial variance, respectively; none of them uniquely indexes recovery of consciousness.

### 3.1 Spectral findings: aperiodic exponent and the 20–35 Hz window

The post-stimulation flattening of the aperiodic exponent at C3, P4, Pz, and CP1 in the R+ group is consistent with a shift in cortical excitation–inhibition (E/I) balance toward enhanced excitability. The aperiodic 1/f slope has been proposed as a mesoscale proxy for E/I balance: a steeper slope reflects greater inhibitory dominance, whereas a flatter slope indicates increased excitatory drive (Gao et al., 2017). In chronic DoC, excessive GABAergic inhibition is considered a hallmark of the suppressed cortical state characteristic of VS/UWS. The observed reduction in the aperiodic exponent is therefore compatible with the primary mechanism of anodal tDCS, which induces subthreshold depolarization of the resting membrane potential and facilitates neuronal firing. Importantly, this EEG pattern was observed only in responders and may be compatible with altered E/I balance, suggesting that the capacity for such modulation may reflect preserved cortical plasticity in a subset of patients with DoC.

The 20–35 Hz window was used as a hypothesis-driven summary of the faster spectral range depicted in the C- and D-type ABCD profiles; it was not treated as an independent ABCD category. An increase in this interval may be compatible with an altered contribution of faster activity within a relatively preserved corticothalamic functional state, but isolated scalp power cannot establish central-thalamic output or thalamocortical connectivity. Cortical activation, arousal and vigilance, medication, residual electromyographic activity, signal-to-noise ratio and broader network processes provide alternative or contributing explanations. The range also spans the conventional beta to low-gamma transition and has not been independently validated as a boundary in its own right. Because the band-limited estimate is derived from the full model fit and therefore includes the aperiodic component, and because the aperiodic exponent also changed in responders, the two spectral findings should not be regarded as independent; an aperiodic-corrected analysis is provided in Supplementary Table S4.

Four electrodes (C3, P4, Pz, and CP1) contributed to clusters in both spectral analyses, within the centro-parietal territory surrounding the stimulation site. As noted above, however, the two measures are not statistically independent, and this spatial correspondence should not be interpreted as convergent evidence from separate mechanisms. Earlier EEG studies of tDCS in disorders of consciousness, which targeted the left dorsolateral prefrontal cortex, reported stimulation-related spectral changes without separating periodic and aperiodic components (Cavinato et al., 2019).

### 3.2 Nonlinear dynamics

The decrease in RQA metrics (RR, DET, L and ENTR) alongside the increase in LZc forms a consistent pattern across the two families of nonlinear measures. In chronic DoC, cortical dynamics have been described as becoming trapped in repetitive, stereotyped patterns characterized by high recurrence and high determinism, a state referred to as “dynamical ruts” (Demertzi et al., 2019). The present observations are compatible with a reduction in that regularity together with a more varied symbolic repertoire (Tononi et al., 2016), although RQA and LZc are both computed from the same filtered time series and are not independent descriptors. A comparable direction of effect is predicted by accounts linking complexity to integrated information, including work on the Perturbational Complexity Index (PCI) (Sarasso et al., 2021; Casali et al., 2013); the correspondence is one of direction only, since PCI is derived from an evoked cortical response and the present measures are not.

Unlike PCI, which requires TMS perturbation, the measures used here are computed from resting-state recordings alone. Resting-state EEG is substantially less resource-intensive than TMS–EEG, which may make RQA and LZc easier to deploy for repeated monitoring, subject to the interpretive limits set out above.

The seven electrodes significant across all four RQA metrics (C3, CP1, CP2, CP6, P4, P8 and Pz) overlap those identified in the spectral analyses. Because all of these measures are derived from the same recordings and are sensitive to overlapping properties of the signal, this spatial correspondence is not independent corroboration; it indicates that the changes were most pronounced in the same centro-parietal region rather than that separate mechanisms converge there (Kang et al., 2020).

### 3.3 Spatial complexity

The increase in 20–35 Hz normalized spatial complexity (NSC) in the R+ group extends the description from electrode-level temporal dynamics to the distribution of spatial variance across the scalp. NSC quantifies how evenly variance is distributed across spatial components; an increase indicates a more even distribution. This measure and its application to the present cohort were reported previously (Zhang et al., 2022) across seven canonical bands, and the present result in the 20–35 Hz window overlaps the beta2 finding reported there.

Interpretation in terms of integration and differentiation, as proposed within Integrated Information Theory, remains a theoretical framing rather than a demonstrated correspondence. The combination of increased 20–35 Hz power and increased NSC in the same window may be compatible with such an account, but the present data cannot distinguish it from alternatives involving changes in vigilance, arousal or signal-to-noise ratio.

### 3.4 Explainable machine learning as a bridge from mechanism to prediction

Two pre-stimulation features supported discrimination between responders and non-responders. Relief ranking, applied within the training data of each resampling fold, converged on the aperiodic exponent and the 20–35 Hz NSC feature, and SHAP values indicated comparable contributions from both. With 42 participants and 10 non-responders these estimates carry wide uncertainty and require external validation before any clinical interpretation. They are not comparable with the NSC-based classifier reported for this cohort previously (Zhang et al., 2022), which used a different feature set, resampling procedure and performance summary.

The agreement between data-driven feature ranking and the hypothesis-driven analyses is descriptive rather than confirmatory, since both were derived from the same dataset. From a clinical perspective, SHAP addresses the interpretability requirement that is critical for decision support in high-stakes, low-sample settings such as DoC. Rather than providing a black-box prediction, the model offers transparent explanations for individual classifications—for example, identifying whether low baseline spatial complexity or a steep 1/f slope contributed to a non-responder prediction. Existing machine learning models in DoC have focused predominantly on diagnostic categorization rather than prediction of neuromodulatory responsiveness; the present framework is a step toward prediction-guided treatment selection, subject to external validation.

### 3.5 Posterior parietal stimulation and the posterior hot zone hypothesis

The “posterior hot zone” hypothesis identifies the temporo-parieto-occipital association cortex, including the precuneus and posterior cingulate cortex, as a candidate substrate for conscious content (Boly et al., 2017). Most tDCS interventions in DoC have instead targeted the left DLPFC, with variable reported efficacy. Stimulation delivered over Pz, a scalp entry point approximating this posterior territory, was followed by spectral, nonlinear and spatial-complexity changes in responders and by no detectable change in non-responders. This dissociation is consistent with the posterior cortex being functionally relevant to the effects of stimulation, but it does not establish it: the measures are not independent of one another, no alternative stimulation site or sham condition was included here, and responder status was defined from the same behavioral assessment used to characterize outcome. The pattern is compatible with accounts that emphasize posterior regions, including IIT and Recurrent Processing Theory (Seth and Bayne, 2022; Seth and Hohwy, 2021; Ye et al., 2020), without providing a test that distinguishes between them.

### 3.6 Limitations and future directions

Several limitations should be emphasized. The study is a single-arm secondary analysis without sham stimulation, so pre-to-post changes cannot be attributed specifically to HD-tDCS; spontaneous fluctuation, repeated assessment and non-specific effects of the treatment context cannot be excluded. The cohort and its clinical outcomes have been reported previously (Zhang et al., 2022), and the present analyses are therefore not independent of that report. All measures are indirect scalp-derived descriptors whose physiological interpretation is inferential. The spectral smoothing bandwidth of ±4 Hz limits the resolution with which narrow low-frequency features can be separated, and the absence of an effect in the 5–9 Hz window should not be read as evidence that no low-frequency change occurred. RQA estimates depend on embedding and threshold choices adopted from prior literature rather than optimized here, and a fixed absolute recurrence threshold is sensitive to signal amplitude. Individual medication records were not available at participant level. Finally, with 42 participants and 10 non-responders, the predictive performance estimates carry wide uncertainty and require external validation. Additionally, CRS-R total scores are ordinal, and equal numerical increases at different points on the scale do not necessarily reflect equivalent clinical improvement. Rasch-transformed scores would provide interval-level measurement and a more clinically meaningful quantification of change (Weaver et al., 2024). Furthermore, measurable electrophysiological changes do not necessarily translate into qualitative neuropsychological improvement observable at the bedside; the clinical significance of the EEG biomarkers reported here therefore requires validation against functional outcomes beyond CRS-R score changes.

Future research should address these limitations through multi-center, sham-controlled trials with extended follow-up, and source-level EEG or concurrent fMRI would be required to determine whether the observed spectral changes have a thalamic contribution. Source-localization techniques such as eLORETA, which can approximate deep-source activity from scalp recordings, could provide an initial estimate of thalamic involvement without requiring additional imaging modalities. Larger-scale self-supervised EEG foundation models are an increasingly important direction and may eventually improve representation transfer across cohorts and acquisition systems (Sümer-Arpak et al., 2026). Their application to neuromodulation in DoC remains constrained by small labeled cohorts, heterogeneous acquisition protocols and the scarcity of external validation; training a high-capacity model *de novo* on 42 participants would risk overfitting and would sacrifice the interpretability that clinical adoption requires. Related work on symbolic and eigen-decomposition-based EEG biomarkers shows how features robust to preprocessing variability can be constructed for clinical classification (Varun et al., 2026), and comparable robustness analyses would be valuable for the measures reported here. Multicenter work should evaluate pretrained EEG encoders under frozen or parameter-efficient adaptation against rigorously tuned conventional models with participant-level external validation. The two features identified by Relief could serve as prospective enrichment biomarkers, subject to such validation.

## 4 Conclusion

This analysis identified responder-specific changes in periodic and aperiodic spectral parameters and in nonlinear EEG descriptors following Pz-targeted HD-tDCS in chronic disorders of consciousness. These associations generate hypotheses about cortical-state and network-level modulation, but they do not directly demonstrate excitation–inhibition rebalancing, thalamocortical loop reinforcement, or engagement of a unique posterior mechanism of consciousness. Two pre-stimulation features supported above-chance discrimination between responders and non-responders, providing a starting point for prospective, externally validated work on precision neuromodulation in this population.

## Data Availability

The raw data supporting the conclusions of this article will be made available by the authors, without undue reservation.
