# Supplementary material for: Multidimensional EEG mechanisms and explainable prediction of responsiveness to posterior parietal HD-tDCS in chronic disorders of consciousness
Source: Front Neurosci. 2026 Sep 14;20:1925503. doi: 10.3389/fnins.2026.1925503 (PMC13617461; doi:10.3389/fnins.2026.1925503)
Supplement: Supplementary file 1 [file Data_Sheet_1.pdf]

Supplementary Material

Multidimensional EEG Mechanisms and Explainable Prediction of Responsiveness to Posterior Parietal HD-tDCS in Chronic Disorders of Consciousness

Xiangyun Zou, Bin Jiang, Chunyun Zhang

Contents

Methods sections 1–9 are cited in the main text as §1–§9.

**Part A**	**Supplementary Methods**
1	Study provenance
2	EEG acquisition and preprocessing
3	Spectral estimation and parameterisation
4	Frequency windows
5	Nonlinear dynamical measures
6	Normalized spatial complexity
7	Statistical analysis
8	Machine-learning pipeline
9	References cited in the Supplementary Methods
**Part B**	**Supplementary Tables**
Table S1	Scope of the present study in relation to Zhang et al. (2022)
Table S2	Participant-level demographic and clinical characteristics
Table S3	Software and numerical parameters
Table S4	Cluster-based permutation tests by canonical frequency band

Part A. Supplementary Methods

All analysis code parameter is provided as Supplementary.

§1. Study provenance

This is a secondary analysis of the clinical and EEG dataset reported by Zhang et al. (2022, *Front Neurosci* 16:903703).

Item	Value	Source
Recruitment sites	Fifth Affiliated Hospital of Zhengzhou University; Zhengzhou Central Hospital, Zhengzhou University	Zhang et al. (2022)
Recruitment period	October 2016 – October 2020	Zhang et al. (2022)
Enrolled	45	Zhang et al. (2022)
Excluded from EEG analysis (signal quality)	3	Zhang et al. (2022)
Analysed	42 (responders 32, non-responders 10)	Present analysis
Original ethics approvals	KY2020024; 201614	Zhang et al. (2022)
Secondary-analysis approval	KYLL-KS-2025153, Ethics Committee of Qilu Hospital of Shandong University (Qingdao)	Present study
Intervention	14 days, twice daily, Pz-targeted 4×1 ring HD-tDCS, 2 mA, 20 min per session	Zhang et al. (2022)
EEG time points	T0 (pre-stimulation), T1 (post-stimulation)	—

§2. EEG acquisition and preprocessing

Acquisition state. Behaviourally monitored wakeful rest. Where required, the CRS-R arousal-facilitation protocol was applied immediately before recording. If a participant closed the eyes for more than a few seconds, or the EEG showed sleep spindles or K-complexes, the recording was terminated, the data discarded, and the arousal protocol and acquisition repeated.

Preprocessing (EEGLAB), in execution order:

1. Band-pass FIR filter, 0.5–40 Hz
2. Notch filter, 48–52 Hz (line noise)
3. Visual inspection; manual rejection of segments containing gross artefacts (movement, electrode pops)
4. Bad-channel identification by two experienced electrophysiologists; spherical-spline interpolation

5. Independent component analysis; removal of ocular, cardiac and myogenic components
6. Re-referencing to the common average
7. Datasets retaining <80% of the recording were treated as invalid

Montage. 30 scalp electrodes (international 10–20 system): Fp1, Fp2, Fpz, F7, F3, Fz, F4, F8, FC5, FC1, FC2, FC6, T7, C3, Cz, C4, T8, CP5, CP1, CP2, CP6, P7, P3, Pz, P4, P8, O1, Oz, O2, POz.

Segmentation differs between the two analysis branches:

Branch	Resampling	Epoch length	Overlap
Spectral (FOOOF, band-limited estimates)	none (native rate)	epoched dataset; effective analysis window yields 0.5-Hz resolution	none
Nonlinear (RQA, LZc)	100 Hz	5 s (500 samples)	2 s

---

§3. Spectral estimation and parameterisation

Power spectral density. FieldTrip `ft_freqanalysis`:

Parameter	Value
`cfg.method`	`mtmfft`
`cfg.taper`	DPSS (Slepian), FieldTrip default for `mtmfft`
`cfg.foilim`	`[1 40]` Hz
`cfg.tapsmofrq`	4 (±4 Hz; 8-Hz smoothing kernel)
`cfg.output`	`pow`, `fooof`, `fooof_peaks`, `fooof_aperiodic` (computed separately)
Frequency resolution	0.5 Hz
Frequency bins	79 (1.0 to 40.0 Hz)
Averaging	across epochs within participant and channel

Spectral smoothing. The ±4 Hz kernel is appropriate for broadband spectral characterisation but limits resolution of narrow or closely spaced spectral features. The 5–9 Hz window is 4 Hz wide and therefore falls entirely within one smoothing kernel, so theta- and alpha-range features cannot be resolved separately within it.

Spectral parameterisation (FOOOF / specparam), via FieldTrip's `fooof` outputs, default settings of the underlying algorithm:

Parameter	Value
Aperiodic mode	fixed (no knee)

Fit range	1–40 Hz
Peak width limits	[0.5, 12] Hz
Maximum number of peaks	unlimited
Minimum peak height	0
Peak threshold	2.0 SD

Aperiodic parameters (offset, exponent) and peak parameters (centre frequency, power, bandwidth) were exported per channel and participant.

Canonical reference: Donoghue et al. (2020), *Nature Neuroscience* 23:1655–1665. FieldTrip implementation: Oostenveld et al. (2011), *Comput Intell Neurosci* 2011:156869.

Spectral outputs:

Output	Content	Units
`pow`	Observed power spectral density	$\mu V^2/Hz$
`fooof`	Full model fit = aperiodic component × peak component	$\mu V^2/Hz$
`fooof_aperiodic`	Fitted aperiodic (1/f) component only	$\mu V^2/Hz$
`fooof_peaks`	Peak component only, expressed relative to the aperiodic fit	dimensionless

Band-limited estimates in the main text are the mean of the full model fit (`fooof`) across the frequency bins of each band and therefore include the aperiodic component. Table S4 reports the corresponding aperiodic-corrected analysis.

---

§4. Frequency windows

Label	Range	Bins (0.5-Hz resolution)	Basis
ABCD-informed low-frequency window	5–9 Hz	9	Annotated range for the B-type profile, Comanducci et al. (2020) Fig. B1, after Schiff (2016)
ABCD-informed higher-frequency window	20–35 Hz	31	Annotated range shared by the C- and D-type profiles, same source
Delta	1–4 Hz	6	Canonical; lower bound set by the 1–40 Hz fit range
Theta	4–8 Hz	8	Canonical
Alpha	8–13 Hz	10	Canonical

Beta	13–30 Hz	34	Canonical
Gamma	30–40 Hz	21	Canonical

The five canonical bands partition the 1–40 Hz range without overlap or omission (6 + 8 + 10 + 34 + 21 = 79 bins). The two ABCD-informed windows overlap the canonical bands (5–9 Hz spans theta into low alpha; 20–35 Hz spans beta into low gamma), so estimates from the two schemes are not independent.

---

§5. Nonlinear dynamical measures

Computed on 100-Hz data, 5-s epochs with 2-s overlap, per channel; epoch-level values averaged within channel and participant.

Recurrence quantification analysis (RQA):

Parameter	Value
Embedding dimension (m)	3
Time delay (τ)	1 sample
Recurrence threshold (ε)	0.5, fixed
Distance metric	Euclidean
Measures extracted	Recurrence rate (RR), determinism (DET), average diagonal line length (L), Shannon entropy of the diagonal line length distribution (ENTR)

Parameter values were adopted from prior applications of RQA to EEG rather than optimised on the present dataset. Methodological reference: Marwan et al. (2007), *Physics Reports* 438:237–329.

Limitation. A fixed absolute recurrence threshold is sensitive to signal amplitude. Recurrence-rate-matched thresholding would be a more conservative alternative and is recommended for future work.

Lempel–Ziv complexity (LZc):

Parameter	Value
Binarisation	epoch mean amplitude as threshold
Sequence length	500 samples (5 s at 100 Hz), identical for every estimate
Aggregation	epoch values averaged within channel and participant

Variant reported	mean-threshold binarisation (`LZc_mean`)

Because all sequences are of equal length, values are comparable across participants and time points without further length normalisation. Median-threshold, midpoint-threshold, k-means binarisation and permutation Lempel–Ziv complexity are also implemented in the supplied code.

Reference: Lempel & Ziv (1976), *IEEE Trans Inf Theory* 22:75–81. Application to consciousness: Schartner et al. (2015), *PLoS One* 10:e0133532; Liu et al. (2023), *BMC Neurol* 23:140.

---

§6. Normalized spatial complexity (NSC)

For each frequency band, the preprocessed data were band-pass filtered into that band and temporal principal component analysis was applied to the channel × time matrix across **all 30 channels** (whole-brain; no regional subdivision), yielding eigenvalues λ_i of the channel covariance matrix.

Normalised contribution of component *i*:

$$p_i = \lambda_i / \Sigma_j \lambda_j$$

Normalized spatial complexity:

$$NSC = -\Sigma_i p_i \ln p_i / \ln N$$

where N is the number of retained components (eigenvalues below 1×10^{-15} were discarded as numerical noise). NSC approaches 0 when variance is concentrated in a single spatial component and 1 when variance is distributed uniformly. Values were computed per epoch and averaged across epochs within participant.

Computed separately for seven bands: delta (1–4), theta (4–8), alpha (8–13), beta (13–30), gamma (30–40), and the two ABCD-informed windows (5–9, 20–35 Hz).

Reference: Jia, Li & Yu (2018), *Scientific Reports* 8:7912.

Relationship to prior work. NSC was applied to this cohort across seven canonical bands in Zhang et al. (2022), where an increase in the beta2 band (20–30 Hz) was reported in responders. The 20–35 Hz result reported here overlaps that finding and is therefore not independent of it.

---

§7. Statistical analysis

Electrode-wise pre- versus post-stimulation comparisons, performed separately within each response group:

Parameter	Value

Test statistic	dependent-samples *t* (`depsamplesT`)
Method	Monte Carlo permutation (`montecarlo`)
Multiple-comparison correction	cluster-based (`correctm = 'cluster'`)
Cluster statistic	`maxsum`
Cluster-forming threshold	α = 0.001
Number of permutations	5000
Tail	two-tailed (`cfg.tail = 0`, `cfg.clustertail = 0`)
Minimum neighbouring channels	not specified (default 0)
Channel adjacency	derived from the 2-D electrode layout via `ft_prepare_neighbours`

Inferential scope. Cluster-based permutation testing licenses inference at the level of the cluster, not the individual electrode. Electrode identities are reported descriptively, to characterise the spatial extent of each cluster.

NSC comparisons. Paired *t* tests within each response group, with Benjamini–Hochberg false discovery rate correction across frequency bands. Significance level α = 0.05, two-tailed.

Software. MATLAB R2024b; EEGLAB; FieldTrip. Exact versions are given in Supplementary Table S3.

---

§8. Machine-learning pipeline

Objective. Classify participants as responders or non-responders using **pre-stimulation (T0) features only**.

Candidate features (8), measured at Pz or at the global level:

1. Aperiodic exponent (Pz)
2. Band-limited power, 20–35 Hz window
3. Determinism (DET)
4. Average diagonal line length (L)
5. Recurrence rate (RR)
6. Shannon entropy of diagonal line lengths (ENTR)
7. Mean Lempel–Ziv complexity (LZc_mean)
8. Normalized spatial complexity, 20–35 Hz window

Validation structure. Nested resampling. The outer loop is leave-one-participant-out; within each outer training set an inner stratified *k*-fold cross-validation is used for model selection.

Order of operations within each outer fold — all steps fitted on the outer training participants only:

1. Standardisation fitted on outer-training data
2. Inner stratified cross-validation for model selection: feature ranking, number of retained features, and classifier hyperparameters
3. Class imbalance addressed within training data only
4. Final fold model fitted on the complete outer training set
5. Single prediction of the held-out participant

The held-out participant contributes to no step of scaling, feature ranking, resampling or hyperparameter optimisation.

Classifier. Support vector machine with radial basis function kernel; hyperparameters selected by Bayesian optimisation within the inner loop.

Performance metrics. Computed from the pooled out-of-fold predictions: sensitivity, specificity, balanced accuracy, overall accuracy, AUC, and the confusion matrix.

Model comparison. The Relief-selected feature set and the full eight-feature set were evaluated under the identical validation framework, so that the contribution of feature selection can be assessed directly.

Interpretation. SHAP values characterise feature contributions. Where SHAP is computed on a model fitted to all participants, that model serves a descriptive purpose only and its apparent performance is not a validated estimate.

---

§9. References cited in these Supplementary Methods

1. Comanducci A, Boly M, Claassen J, et al. Clinical and advanced neurophysiology in the prognostic and diagnostic evaluation of disorders of consciousness: review of an IFCN-endorsed expert group. *Clin Neurophysiol.* 2020;131:2736–2765. doi:10.1016/j.clinph.2020.07.015

2. Donoghue T, Haller M, Peterson EJ, et al. Parameterizing neural power spectra into periodic and aperiodic components. *Nat Neurosci.* 2020;23:1655–1665. doi:10.1038/s41593-020-00744-x

3. Jia H, Li Y, Yu D. Normalized spatial complexity analysis of neural signals. *Sci Rep.* 2018;8:7912. doi:10.1038/s41598-018-26329-0

4. Lempel A, Ziv J. On the complexity of finite sequences. *IEEE Trans Inf Theory.* 1976;22:75–81.

5. Liu Y, Zeng W, Pan N, Xia X, Huang Y, He J. EEG complexity correlates with residual consciousness level of disorders of consciousness. *BMC Neurol.* 2023;23:140. doi:10.1186/s12883-023-03167-w

6. Marwan N, Romano MC, Thiel M, Kurths J. Recurrence plots for the analysis of complex systems. *Phys Rep.* 2007;438:237–329. doi:10.1016/j.physrep.2006.11.001

7. Oostenveld R, Fries P, Maris E, Schoffelen JM. FieldTrip: open source software for advanced analysis of MEG, EEG, and invasive electrophysiological data. *Comput Intell Neurosci.* 2011;2011:156869.

8. Schartner M, Seth A, Noirhomme Q, et al. Complexity of multi-dimensional spontaneous EEG decreases during propofol induced general anaesthesia. *PLoS One.* 2015;10:e0133532. doi:10.1371/journal.pone.0133532

9. Schiff ND. Mesocircuit mechanisms underlying recovery of consciousness following severe brain injuries: model and predictions. In: Monti MM, Sannita WG, eds. *Brain Function and Responsiveness in Disorders of Consciousness.* Springer; 2016:195–204. doi:10.1007/978-3-319-21425-2_15

10. Sümer-Arpak E, Saini R, Das Chakladar D, Varun SK, Simistira Liwicki F. The current status of foundation models in decoding inner speech from non-invasive brain signals: a mini review. *Front Hum Neurosci.* 2026;20:1838064. doi:10.3389/fnhum.2026.1838064

11. Zhang C, Han S, Li Z, et al. Multidimensional assessment of electroencephalography in the neuromodulation of disorders of consciousness. *Front Neurosci.* 2022;16:903703. doi:10.3389/fnins.2022.903703

12. Varun SK, Bollu TKR, Panwar S, Agarwal P, Lin CT, Antonakakis M, Zervakis M. n-cylindrical Symbolic Response, a standalone and synergistic biomarker for Epilepsy diagnosis on EEG modality. *IEEE J Biomed Health Inform.* 2025. doi:10.1109/JBHI.2025.3635229

Part B. Supplementary Tables

Supplementary Table S1. Scope of the present study in relation to Zhang et al. (2022)

The cohort, the HD-tDCS intervention and the CRS-R outcome assessments are common to both studies. The table below sets out which analyses appear in each.

Analysis	Zhang et al. (2022)	Present study
Conventional-band relative power (Welch)	Reported	Not repeated
PLV-based graph-theoretical measures	Reported	Not repeated
Normalized spatial	Seven	Same measure, applied additionally to

complexity (NSC)	canonical bands, whole-brain	the two ABCD-informed windows (5–9 Hz, 20–35 Hz); the 20–35 Hz result overlaps the beta2 (20–30 Hz) finding reported previously
Classification of response	Seven NSC features	Eight candidate features, one of which is the NSC feature above; Relief ranking and SHAP added
FOOOF periodic–aperiodic parameterisation	Not examined	Reported here
Recurrence quantification analysis (RQA)	Not examined	Reported here
Lempel–Ziv complexity (LZc)	Not examined	Reported here
Canonical-band analysis of aperiodic-corrected periodic power	Not examined	Reported here (Table S4)

Because the two studies draw on the same participants and the same clinical outcomes, the analyses reported here are not statistically independent of those in the earlier report.

Supplementary Table S2. Participant-level demographic and clinical characteristics

(a) Baseline (T0)

No.	Sex	Age	Dx	Aud	Vis	Mot	Oro	Com	Aro	Total
1	M	44	MCS-	2	2	2	1	0	2	9
2	M	37	VS	1	1	2	0	0	2	6
3	M	62	MCS-	2	3	3	1	0	2	11
4	M	48	VS	1	1	2	1	0	1	6
5	F	52	MCS-	2	2	2	1	0	1	8
6	F	65	VS	1	1	2	0	0	2	6
7	M	30	MCS-	1	2	2	1	0	1	7
8	M	28	MCS-	2	3	2	2	0	2	11
9	M	51	VS	2	1	2	0	0	1	6
10	M	51	MCS-	1	1	5	1	0	1	9
11	F	40	VS	1	1	2	1	0	1	6
12	M	36	VS	1	1	2	1	0	1	6
13	F	52	MCS-	2	2	2	1	0	2	9
14	F	60	MCS-	2	3	2	1	1	2	11
15	M	8	MCS-	1	2	2	1	0	1	7
16	M	54	VS	1	1	2	1	0	1	6
17	F	51	MCS-	1	2	2	1	0	1	7
18	M	48	VS	1	1	3	0	0	1	6
19	M	52	MCS-	2	3	3	1	0	1	10

20	M	38	MCS-	2	1	3	1	0	1	8
21	F	38	MCS-	1	1	3	2	0	1	8
22	F	54	MCS-	1	1	3	1	0	1	7
23	F	62	MCS-	1	0	3	1	0	1	6
24	F	72	MCS+	3	2	3	1	1	2	12
25	M	54	VS	0	0	1	0	0	1	2
26	M	14	MCS-	2	3	2	1	0	2	10
27	M	41	VS	1	0	2	0	0	1	4
28	M	52	VS	0	1	1	0	0	0	2
29	M	55	MCS-	2	2	2	1	0	1	8
30	M	63	MCS-	3	2	2	1	0	2	10
31	F	60	MCS+	3	4	4	1	1	3	16
32	M	70	MCS+	3	2	4	1	1	2	13
33	F	66	MCS-	1	2	2	1	0	1	7
34	M	67	MCS-	2	3	3	1	0	2	11
35	F	69	MCS+	3	3	2	1	1	2	12
36	M	65	MCS-	1	3	4	1	0	1	10
37	M	60	VS	0	1	1	0	0	1	3
38	M	52	MCS+	4	4	4	1	1	2	16
39	M	54	VS	0	0	1	1	0	1	3
40	M	61	MCS-	1	2	1	1	0	1	6
41	M	67	MCS-	2	3	3	1	0	1	10
42	F	62	MCS+	3	4	5	1	1	3	17

(b) After 14 days of Pz-targeted HD-tDCS (T1)

No.	Sex	Age	Dx	Aud	Vis	Mot	Oro	Com	Aro	Total	Δ	Group
1	M	44	MCS-	2	2	3	1	0	2	10	+1	R+
2	M	37	VS	1	1	2	0	0	2	6	+0	R–
3	M	62	EMCS	4	5	6	2	2	3	22	+11	R+
4	M	48	VS	1	1	2	1	0	1	6	+0	R–
5	F	52	MCS+	3	4	4	1	0	2	14	+7	R+
6	F	65	MCS-	1	2	2	1	0	2	8	+2	R+
7	M	30	MCS-	3	3	3	1	1	2	13	+6	R+
8	M	28	MCS+	3	3	2	2	1	2	13	+2	R+
9	M	51	MCS-	2	2	2	1	0	1	8	+2	R+
10	M	51	MCS-	2	3	5	1	0	2	13	+4	R+
11	F	40	VS	1	1	2	1	0	2	7	+0	R–
12	M	36	VS	1	1	2	1	0	2	7	+1	R+
13	F	52	MCS+	3	4	5	1	1	2	16	+8	R+
14	F	60	MCS+	4	3	4	2	1	3	17	+6	R+
15	M	8	MCS+	3	3	5	2	1	2	16	+9	R+
16	M	54	MCS-	1	1	2	1	0	1	6	+0	R–
17	F	51	MCS+	3	3	3	2	1	2	14	+8	R+
18	M	48	MCS-	1	2	3	0	0	1	7	+1	R+
19	M	52	MCS-	2	3	4	1	0	2	12	+2	R+
20	M	38	MCS+	3	3	4	1	1	2	14	+6	R+
21	F	38	MCS-	1	2	4	2	0	2	11	+3	R+
22	F	54	MCS-	2	3	3	1	0	1	10	+3	R+
23	F	62	MCS-	1	0	4	1	0	2	8	+2	R+
24	F	72	MCS+	3	3	3	1	1	2	13	+1	R+
25	M	54	VS	0	0	1	0	0	1	2	+0	R–
26	M	14	MCS-	2	3	3	1	1	2	12	+2	R+

27	M	41	VS	1	0	2	0	0	1	4	+0	R−
28	M	52	VS	0	1	1	0	0	0	2	+0	R−
29	M	55	MCS-	2	3	3	1	0	2	11	+3	R+
30	M	63	MCS-	3	2	2	1	0	2	10	+0	R−
31	F	60	MCS+	4	4	4	1	1	3	17	+1	R+
32	M	70	MCS+	4	4	5	2	1	2	18	+5	R+
33	F	66	MCS-	2	3	2	1	2	2	12	+3	R+
34	M	67	MCS+	3	3	3	1	1	2	13	+2	R+
35	F	69	MCS+	4	3	2	1	1	2	13	+1	R+
36	M	65	MCS-	2	3	4	1	0	1	11	+1	R+
37	M	60	VS	0	1	1	0	0	1	3	+0	R−
38	M	52	EMCS	4	5	6	3	2	3	23	+7	R+
39	M	54	VS	0	0	2	1	0	2	5	+2	R+
40	M	61	MCS-	1	2	2	1	0	2	8	+2	R+
41	M	67	MCS-	2	3	3	1	0	1	10	+0	R−
42	F	62	EMCS	4	5	6	3	1	2	21	+4	R+

Aud, auditory; Vis, visual; Mot, motor; Oro, oromotor/verbal; Com, communication; Aro, arousal. VS, vegetative state; MCS−/MCS+, minimally conscious state minus/plus; EMCS, emergence from MCS. R+, responder (32); R−, non-responder (10).

Etiology. Stroke, n = 28; traumatic brain injury, n = 12; anoxic encephalopathy, n = 2.

Medications. No participant received antiepileptic drugs, sedatives, or other agents known to alter cortical excitability during the assessment or stimulation period; use of such medication was an exclusion criterion. Individual dose-level records were not retained in the original study database.

Dx, clinical diagnosis; Δ, change in total CRS-R score.

Supplementary Table S3. Software and numerical parameters

(a) Software

Component	Software	Version
Preprocessing	EEGLAB (MATLAB)	25.1
Spectral estimation, cluster statistics	FieldTrip (MATLAB)	—
Numerical environment	MATLAB	R2024b
Spectral parameterisation	FOOOF (specparam), via FieldTrip `fooof` outputs	—
Machine learning	Python: scikit-learn, imbalanced-learn, SHAP	—

(b) Preprocessing

Parameter	Value
Band-pass filter	FIR, 0.5–40 Hz

Notch filter	48–52 Hz
Artefact handling	manual rejection of gross artefacts; ICA removal of ocular, cardiac, myogenic components
Bad channels	identified by two electrophysiologists; spherical-spline interpolation
Reference	common average
Minimum retained data	≥80% of the recording; ≥15 min artefact-free per participant
Sampling rate	1000 Hz (native); 100 Hz for nonlinear analyses
Epoching (nonlinear branch)	5 s (500 samples), 2 s overlap

(c) Spectral estimation and parameterisation

Parameter	Value
`cfg.method`	`mtmfft`
`cfg.taper`	DPSS (Slepian)
`cfg.foilim`	[1 40] Hz
`cfg.tapsmofrq`	4 (i.e. ±4 Hz, an 8-Hz smoothing kernel)
Frequency resolution	0.5 Hz
Aggregation	per epoch, averaged within participant and channel
FOOOF aperiodic mode	fixed (no knee)
FOOOF fit range	1–40 Hz
FOOOF peak width limits	[0.5 12] Hz
FOOOF maximum number of peaks	unlimited
FOOOF minimum peak height	0
FOOOF peak threshold	2.0 SD

(d) Nonlinear measures

Parameter	Value
RQA embedding dimension (m)	3
RQA time delay (τ)	1 sample
RQA recurrence threshold (ε)	0.5, fixed
RQA distance metric	Euclidean
LZc binarisation	epoch mean amplitude
LZc sequence length	500 samples, identical for every estimate
NSC components retained	eigenvalues > 1×10^{-15}
NSC normalisation	Shannon entropy of normalised eigenvalues ÷ ln N

(e) Cluster-based permutation testing

Parameter	Main analyses (Figs 2–4)	Canonical-band analysis (Table S4)
`cfg.correctm`	cluster	cluster
`cfg.clusteralpha` (cluster-forming)	0.001	0.001
`cfg.alpha` (cluster significance)	0.025	0.025
`cfg.tail` / `cfg.clustertail`	0 (two-tailed)	0 (two-tailed)
`cfg.numrandomization`	5000	5000
`cfg.minnbchan`	0	0
Neighbour definition	`ft_prepare_neighbours, method = 'distance'`	`ft_prepare_neighbours, method = 'triangulation'`
Cluster statistic	maxsum	maxsum
Power quantity tested	mean of the fitted FOOOF model spectrum (includes the aperiodic component)	$\log_{10}$(periodic ÷ own aperiodic component), dimensionless

The two analyses differ in the cluster significance level, the number of permutations, the neighbour definition and the power quantity tested. Estimates from the two columns are therefore not directly comparable.

(f) NSC statistics

Parameter	Value
Test	paired t-test, per frequency band
Multiplicity correction	Benjamini–Hochberg FDR across the seven bands
α	0.05, two-tailed

Supplementary Table S4. Cluster-based permutation tests of aperiodic-corrected periodic power, by canonical frequency band

Band	Group	Direction	Cluster statistic	Monte Carlo P	Channels (n)	Mean dz	Electrodes
delta	R−	post > pre	17.33	0.010*	5	1.10	P4, T8, FC2, FC6, CP6
delta	R−	post > pre	5.25	0.063	2	0.83	F7, FC5
delta	R−	post > pre	2.37	0.167	1	0.75	Fpz

delta	R+	post > pre	2.65	0.126	1	0.47	Pz
delta	R+	post > pre	2.15	0.232	1	0.38	T7
theta	R−	post > pre	5.34	0.068	2	0.84	F8, FC6
theta	R−	post > pre	3.00	0.089	1	0.95	F7
theta	R−	post > pre	2.52	0.114	1	0.80	Fpz
theta	R+	post > pre	27.60	0.014*	11	0.44	Fp1, Fp2, F4, O1, F7, F8, T7, P7, Fz, FC5, FC2
theta	R+	post > pre	8.31	0.052	3	0.49	P4, O2, P8
beta	R−	post < pre	-4.87	0.056	2	-0.77	Cz, FC2
beta	R+	post < pre	-6.98	0.045	3	-0.41	P4, P8, CP6
beta	R+	post < pre	-4.91	0.059	2	-0.43	Cz, FC1
beta	R+	post < pre	-2.08	0.198	1	-0.37	Fp2
gamma	R−	post > pre	2.87	0.022*	1	0.91	T8
gamma	R−	post > pre	2.40	0.048	1	0.76	T7

Within-group comparisons (post minus pre) used dependent-samples t-tests with cluster-based permutation correction (5000 permutations; cluster-forming threshold 0.001; two-tailed cluster significance $\alpha = 0.025$; channel adjacency by triangulation, mean 6.3 neighbours). Periodic power was quantified as the dimensionless ratio of the fitted spectrum to each participant's own aperiodic component, on a $\log_{10}$ scale, so these estimates are independent of the aperiodic exponent. All clusters are listed, including non-significant ones; * marks clusters significant at $\alpha = 0.025$. *d*z is the mean within-cluster paired Cohen's effect size. R+, responders (n = 32); R−, non-responders (n = 10).

A band × group combination without an entry (the alpha band in both groups, and the higher-frequency ranges in responders) indicates that no electrode exceeded the cluster-forming threshold, not that the comparison was omitted. Electrode identities

describe the spatial extent of each cluster; cluster-based permutation testing licenses inference at the cluster level rather than at individual electrodes.
